# A review of the genus *Metalype* Klapálek, with descriptions of three new species from China (Trichoptera, Psychomyiidae)

**DOI:** 10.3897/zookeys.656.10738

**Published:** 2017-02-14

**Authors:** Shuang Qiu, John C. Morse, Yun-jun Yan

**Affiliations:** 1 College of Life Science and Technology, Huazhong University of Science and Technology, Wuhan, Hu-bei Province, People’s Republic of China; 2 Department of Plant and Environmental Sciences, Clemson University, Clemson, South Carolina, 29634-0310, USA; 3 College of Life Science and Technology, Huazhong University of Science and Technology, Wuhan, Hu-bei Province, People’s Republic of China

**Keywords:** Annulipalpia, caddisfly, east Palearctic Region, Oriental Region

## Abstract

Three new species of *Metalype* from China, *Metalype hubeiensis* Qiu & Morse, **sp. n.**, *Metalype shexianensis* Qiu & Morse, **sp. n.**, and *Metalype truncata* Qiu & Morse, **sp. n.**, are described and illustrated. *Metalype uncatissima* (Botosaneanu, 1970) is reported from China for the first time. The differences between genus *Metalype* and genus *Psychomyia* are discussed and four *Psychomyia* species are transferred to *Metalype*: *Metalype holzenthali* (Schmid, 1997); *Metalype klapaleki* (Malicky, 1995a); *Metalype kumari* (Schmid, 1997); and *Metalype nithaiah* (Malicky, 2014). A key to the males of *Metalype* species of the world is provided.

## Introduction

Knowledge of the Chinese Trichoptera fauna was limited before the mid-1900s, described solely by foreign scholars (Morse et al. 1994). It has increased considerably since the 1980s, mostly due to the work of Chinese scientists. There were only 530 Chinese species known by 1990 (Yang et al. 2005), but 1267 Chinese species were described by the middle of 2014 (Yang et al. 2016). However, records of Psychomyiidae increased from 19 species to only 26 species in that interval; this number is relatively small compared to the number of Psychomyiidae species known from the Oriental and East Palearctic Regions (405 spp., Morse unpublished data) and from adjacent countries (e.g., 73 spp. in India, 58 in Thailand, 35 in Vietnam; Morse unpublished data). Schmid (1984) estimated that there are actually 40,000 caddisfly species in southwestern Asia, although this estimate has been questioned by Malicky (1993a). Thus, this study is part of a continuing effort to document the Chinese caddisfly fauna that is mostly unknown to science, focusing here on *Metalype* of Psychomyiidae.

**Figure 1.**
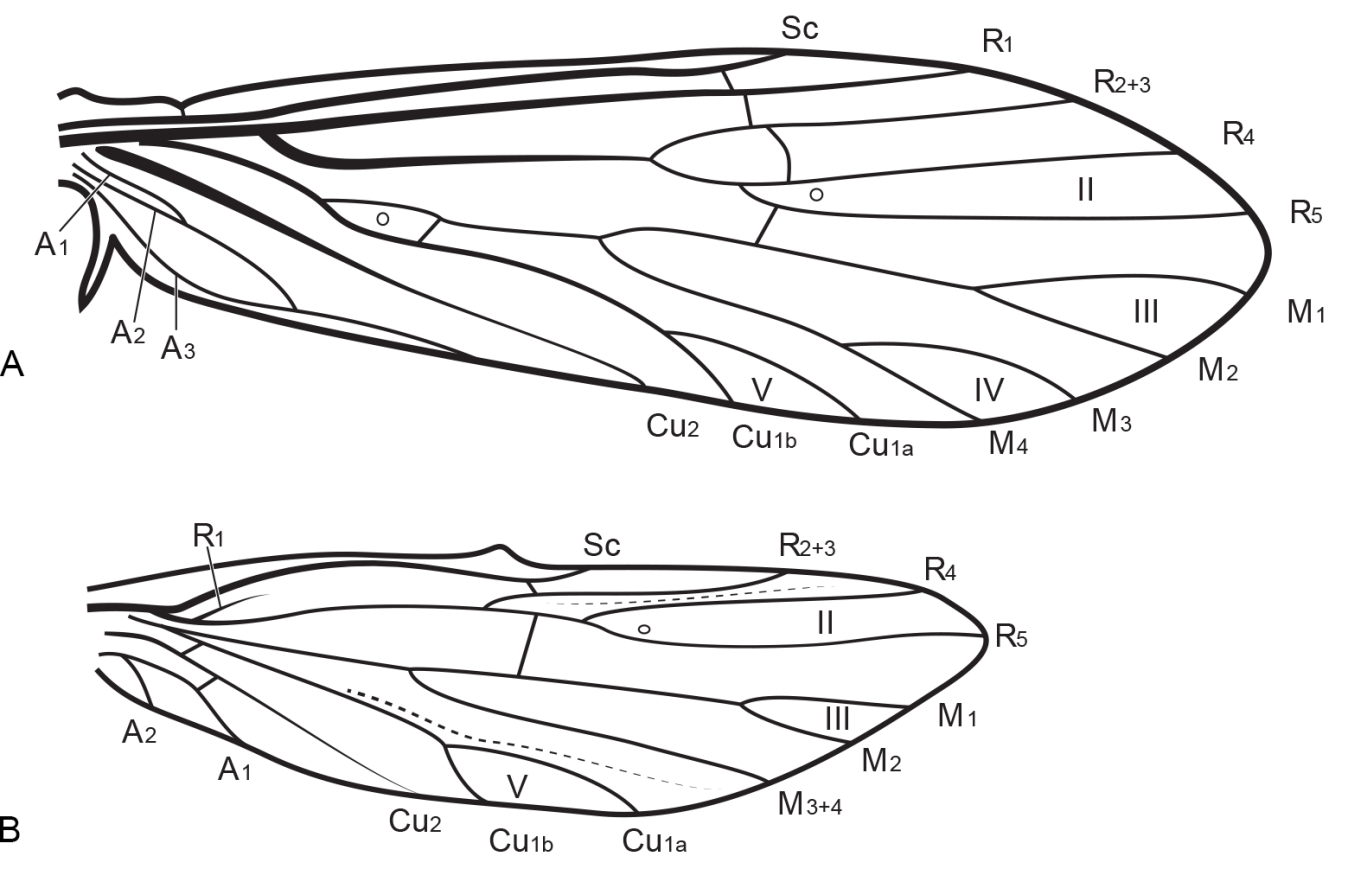
Wing venation of *Metalype truncata* sp. n., right wings, dorsal. **A** forewing **B** hind wing.

The genus *Metalype* was established by Klapálek (1898). For more than 100 years, it contained only the type species *Metalype fragilis* (Pictet, 1834). Wing venation (Fig. 1) and male genitalia of *Metalype* are very similar to those of *Psychomyia* Latreille, 1829 (in Cuvier 1829; type species *Psychomyia annulicornis* Pictet, 1834, selected by Ross 1944, synonym of *Psychomyia pusilla* Fabricius, 1781). Malicky (1995a) suggested that *Metalype* is a synonym of *Psychomyia*. Schmid (1997) treated *Metalype fragilis* as a *Psychomyia* species and included it in his *Psychomyia mahayinna* species group (“*mahayinna* Group”) with six other *Psychomyia* species; he suggested that this group is the oldest lineage of *Psychomyia*. Li and Morse (1997) completed a phylogenetic analysis of Psychomyiidae and concluded that *Metalype* is a monophyletic genus closely related to *Psychomyia* and *Paduniella* Ulmer, 1913 (type species *Paduniella semarangensis* Ulmer, 1913, monotypic), these three genera collectively constituting the subfamily Psychomyiinae. Li and Morse (1997) also listed characters supporting the monophyly of *Metalype* and transferred three *Psychomyia* species to *Metalype*. Later, they indicated that *Metalype* and *Psychomyia* are sister genera, and *Metalype* + *Psychomyia* is the sister lineage to *Paduniella* (Li and Morse 1998). However, some *Metalype* species are still considered to belong in *Psychomyia* by some authors (Robert 2002, Mey and Nozaki 2006, Waringer and Graf 2011, Malicky 2014). Frandsen et al. (2016) concluded that Psychomyiinae is monophyletic, but in addition, they included the genus *Lype* McLachlan, 1878 (type species *Lype phaeopa* (Stephens, 1836), selected by Ross 1944) as sister to *Paduniella* in their phylogeny of this subfamily.

In Asia, *Metalype* species have been reported from Japan (Mey and Nozaki 2006, Nozaki and Nakamura 2007), Korea (Botosaneanu 1970), Nepal (Malicky 1995b), Pakistan (Schmid 1961), and Russia (Levanidova et al. 1995), but not from China (Yang et al. 2016); this apparent absence may have resulted from a lack of studies, or *Metalype* species are recognized in China as species of *Psychomyia*. For example, *Psychomyia nithaiah* Malicky, 2014 was described from Taiwan, but it is probably a *Metalype* species because it is very similar to *Metalype uncatissima* (Botosaneanu, 1970). In this article, we report four *Metalype* species from China, with three of them new to science. We also discuss the differences between *Metalype* and *Psychomyia* species. A key to males of *Metalype* species of the world is also provided.

## Methods

The three new species were first described in Dr Li You-wen’s dissertation (Li 1998), but their names were explicitly excluded from availability under Article 8 of the 3^rd^ edition of the International Code of Zoological Nomenclature (International Commission on Zoological Nomenclature 1985). However, Dr Li deposited his material in the Clemson University Arthropod Collection (CUAC), Clemson, South Carolina, USA; and Department of Plant Protection, Nan-jing Agriculture University (NJAU), Nan-jing, People’s Republic of China (PRC). Here these species are described based on those specimens to make the names available.

Specimens were collected with ultraviolet light traps during 1990–1993 and were preserved in 80% ethanol. The sampling sites are listed in Table 1, with original label names and modern or corrected Chinese names. Holotypes of the new species are deposited in NJAU, paratypes are deposited in NJAU and CUAC. The specimens of *Psychomyia klapaleki* Malicky, 1995a and *Metalype fragilis* were loaned by Dr Hans Malicky from his personal collection in Lunz am See, Austria.

**Table 1.** Locations of *Metalype* species from China.

Species	Province	County	[Geographic coordinate]	Notes	Elevation
*Metalype hubeiensis*	Hu-bei Province 湖北省	[Jing-shan-xian] (Jin-shan-xian) 京山县	[31°16.74'N; 113°12.20'E]	[San-yang Town], Da-fu-shui [三阳镇,] 大富水	90 m
*Metalype shexianensis*	An-hui Province 安徽省	She-xian 歙县	[30°1.19'N; 118°17.84'E]	Yang-jia-tan, Feng-yuan-shui 杨家坦, 丰源水	215 m
			[30°5.94'N; 118°21.54'E]	Yan-yuan Town, Huang-bai-shan Village 岩源镇, 黄柏山村	[717 m]
*Metalype truncata*	Si-chuan Province 四川省	[Jiu-zhai-gou-xian] (Nan-ping-xian) [九寨沟县] (南坪县)	[33°16.02'N; 103°55.08'E]	Jiu-zhai-gou [National Park] 九寨沟[国家公园]	2000 m
		[Du-jiang-yan-shi] (Guan-xian) [都江堰市] (灌县)	[30°53.90'N; 103°34.37'E]	Qing-cheng-shan Town, Wei-jiang-he 青城山镇,味江河	930 m
*Metalype uncatissima*	Hei-long-jiang Province 黑龙江省	Shang-zhi-xian 尚志县	[45°16.40'N; 127°30.26'E]	Mao-er-shan Town, A-shi River 帽儿山镇, 阿什河	300 m
			[44°39.33'N; 128°13.90'E]	Wei-he Town, Yu-lin Tree Farm 苇河镇, 榆林林场	380 m
		Tie-li-shi 铁力市	[46°37.58'N; 129°7.29'E]	Lang-xiang Town, Ba-lan Farm 朗乡镇, 巴兰农场	160 m
		Yi-chun-shi 伊春市	[48°37.09'N; 129°32.96'E]	Wu-yi-ling, Wu-yun River 乌伊岭, 乌云河	160 m

[ ] = information that was not written on the original labels, including modern name or correctly spelled name; ( ) = abandoned name, or name wrongly spelled on the original labels.

Specimens are all preserved in 75%–100% ethanol. Abdomens of a few individuals were removed and water-bath heated in 10% KOH for a few minutes to remove muscle and other non-chitinous tissues for illustration. Specimens were observed under a dissecting microscope. An eyepiece with a grid was used to prepare pencil templates of the various views. The templates were traced with the vector graphics software Adobe Illustrator® (version 19.0.0, 64-bit).

For the specimens that were collected during 1990–1993, no geographical coordinates were taken by GPS at that time. We tried to find the most probable sampling sites based on the location names and descriptions of original labels, and obtained the geographical coordinates from Google Earth (Version 7.1.7.2600). The elevation of one site: An-hui Province, She County, Yan-yuan Town, Huang-bai-shan Village, was missing, so the elevation of this site was also obtained from Google Earth. Elevations of all other sites were obtained from the labels. Modern Chinese names and geographical coordinates of sampling sites were confirmed by Prof Sun Chang-hai (Sun C-h) of Nan-jing Agriculture University.

Terminology for wing venation (Fig. 1) follows that of Schmid (1998). Terminology for male genitalia follows Nielson (1957) except that the pair of flat processes beyond the superior appendages are called “Tergites IX+X” (Ross 1938) and the apical portion of the phallus is called a “phallicata” (“phalicata” [sic], Ross 1956). Terminology for larvae follows Wiggins (1996).

## Results

### Metalype hubeiensis

Taxon classificationAnimaliaTrichopteraPsychomyiidae

Qiu & Morse sp. n.

http://zoobank.org/5320A02D-CC87-4ACE-BD2F-0A48DDCBCBD9

Fig. 2A–E

Metalype hubeiensis Li, 1998: 223–224, figs 11.21–11.24, *nomen nudum*.

#### Type locality.

PRC, Hu-bei Province: Jing-shan County, tributary of Da-fu-shui River, 50 km NW of Ying-cheng downtown, 31°16.74'N; 113°12.20'E, 90 m, 17 July 1990, collector JC Morse.

#### Type specimen.

**Holotype.** Male, in 75% ethanol, in cotton-stoppered microvial inside screwcap vial. Original label: “Hú běi, Jīn-shān-xiàn, 50 KM N.W. of Yīn-chéng, Trib. of Dà-fù-shǔi, 17 July 1990, 90 M elev., coll. Morse” “Metalype hubeiensis Holotype Li & Morse”. Deposited in NJAU.

**Paratypes.** Same data as holotype, 5 males (4 in CUAC, 1 in NJAU). Original label: “Hú běi, Jīn-shān-xiàn, 50 KM N.W. of Yīn-chéng, Trib. of Dà-fù-shǔi, 17 July 1990, 90 M elev., coll. Morse” “Psychomyia sp. 7 鉴定者” [genus and species identity handwritten, Chinese characters = “Identifier”] “Metalype hubeiensis sp. n. paratype Li & Morse” [author names handwritten]. Also, a red paper tag without writing. Deposited in NJAU.

#### Diagnosis.

**Figure 2.**
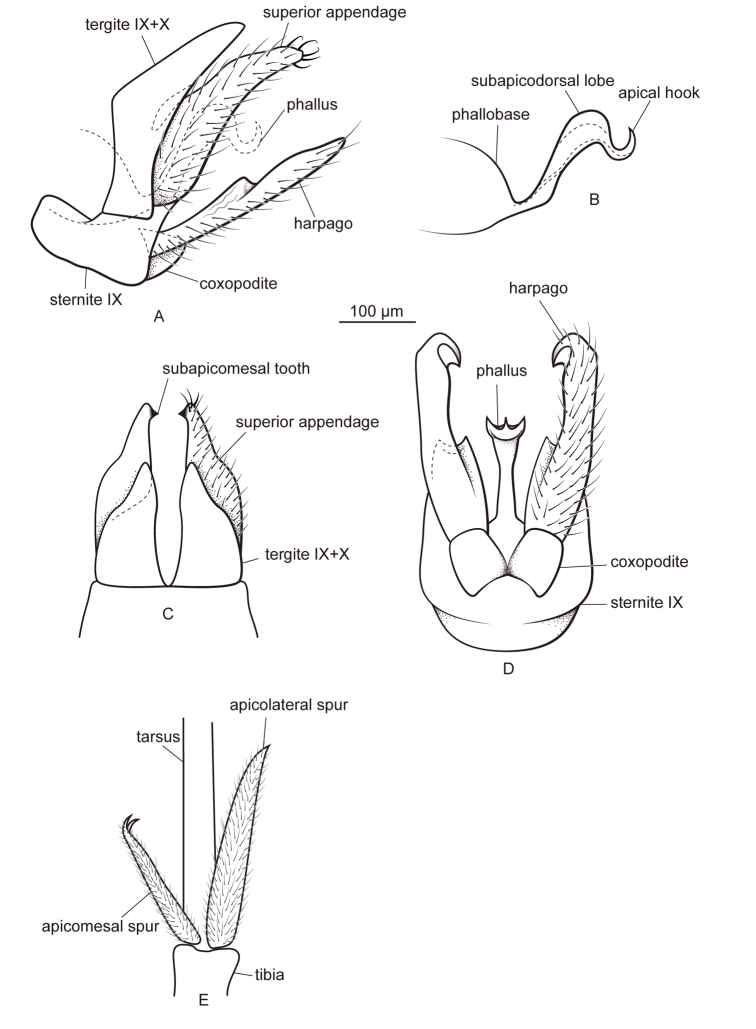
Male of *Metalype hubeiensis* sp. n. **A** genitalia, left lateral **B** phallus, left lateral **C** genitalia, dorsal **D** genitalia, ventral **E** apical spurs of right hind leg, ventral.

**Figure 3.**
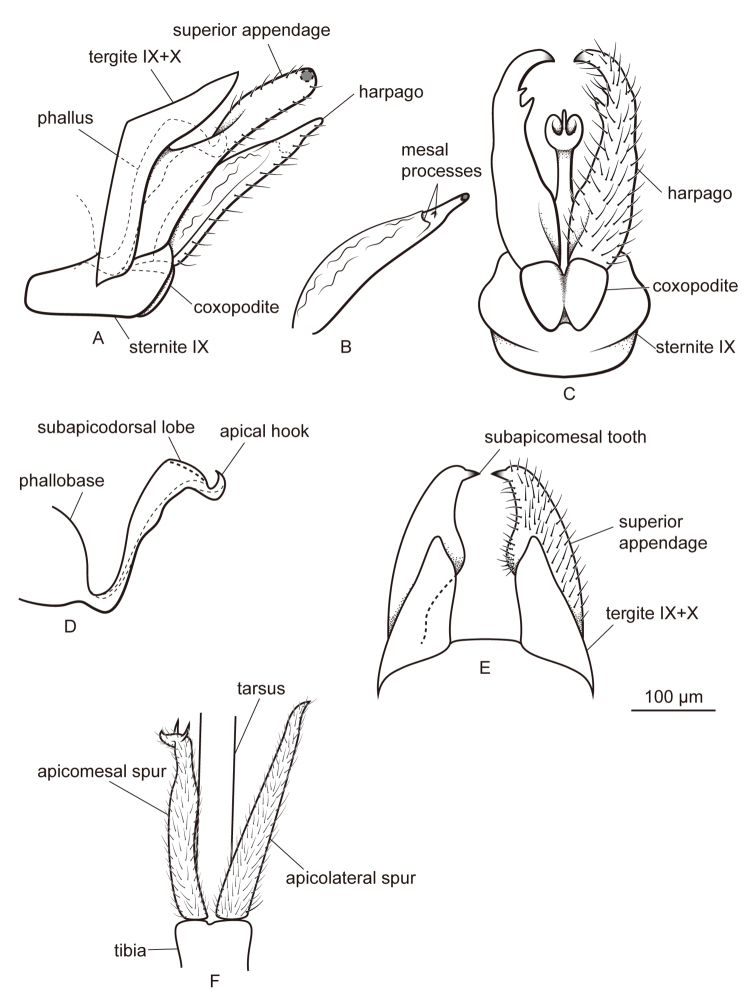
Male of *Metalype shexianensis* sp. n. **A** genitalia, left lateral **B** right harpago, mesal **C** genitalia, ventral **D** phallus, left lateral **E** genitalia, dorsal **F** apical spurs of right hind leg, ventral.

**Figure 4.**
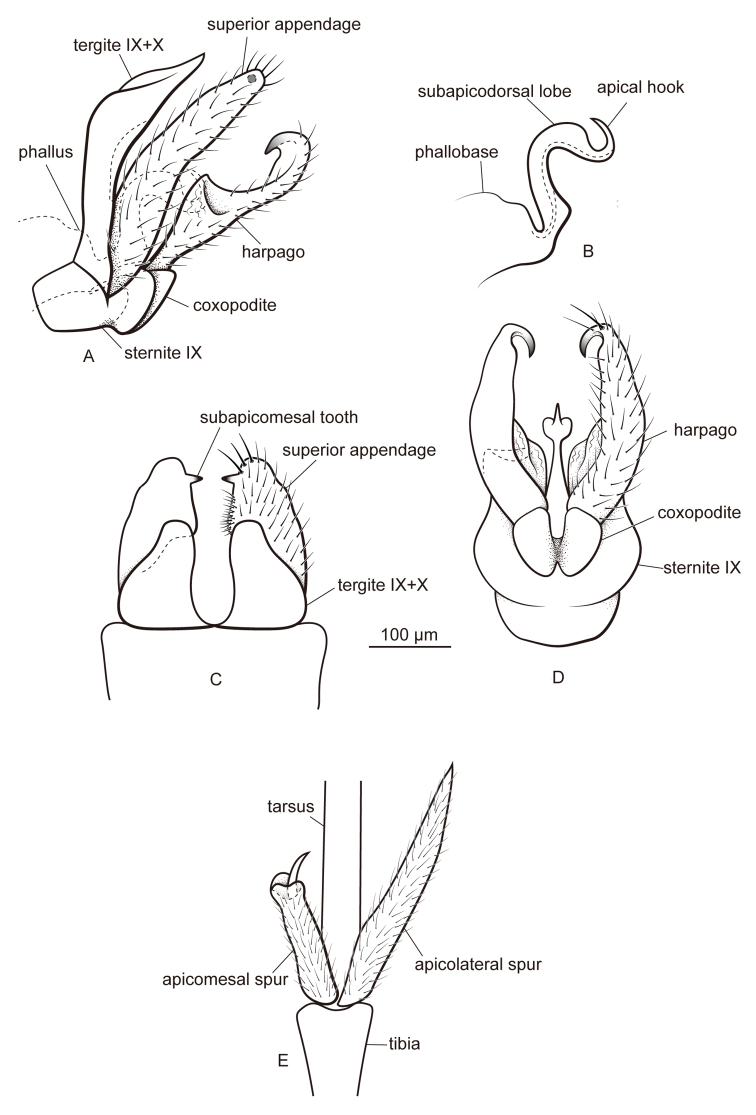
Male of *Metalype truncata* sp. n. **A** genitalia, left lateral **B** phallus, left lateral **C** genitalia, dorsal **D** genitalia, ventral **E** apical spurs of right hind leg, ventral.

This species resembles *Metalype truncata* sp. n. The differences are as follows: (1) The apicomesal spur on each hind leg of *Metalype hubeiensis* is curved mesad and forked apically (Fig. 2E; the apicomesal spur on each hind leg of *Metalype truncata* is truncate apically, with a few lobes and an acute process, Fig. 4E); (2) in ventral view the coxopodites of *Metalype hubeiensis* are fused with each other basally (Fig. 2D; in ventral view the coxopodites are fused with each other for more than half of their length in *Metalype truncata*, Fig. 4D); (3) in lateral view the harpagones of *Metalype hubeiensis* are slightly expanded in the middle dorsally, each less than two times as wide as the basal part (Fig. 2A; in lateral view the harpagones of *Metalype truncata* are strongly expanded in the middle dorsally, each more than two times as wide as the basal part, Fig. 4A); and (4) in ventral view the harpagones of *Metalype hubeiensis* are hooked mesodorsad (Fig. 2D; in ventral view the harpagones of *Metalype truncata* are hooked mesad, Fig. 4D).

#### Description.

**Male.** Forewings each 3.4–3.9 mm (n = 5). Compound eyes black, body yellow. Apicomesal spur of each hind leg slightly curved mesad and forked apically.

**Genitalia.** In lateral view tergites IX+X wide basally, in dorsal view each half triangular and slightly narrowed laterally at two-thirds distance from base. In lateral view superior appendages digitate, wide basally and gradually narrowed from base to apex; in dorsal view central part slightly concave laterally, setose and with few stout and curved setae at apex; each with subapicomesal tooth short, about as long as wide. In ventral view sternite IX slightly expanded posteriorly. In lateral view coxopodites triangular, in ventral view subrectangular and fused with each other only basally. In lateral and ventral views, harpagones each weakly sclerotized and slightly expanded mesodorsad at mid length, in ventral view slightly curved mesad and strongly hooked mesad apically, with harpagonal hook stout and its mesal edge membranous, slightly sclerotized at apex. In lateral view phallus with two major curves, both curves greater than 90°, phallobase expanded, phallicata with pair of round subapicodorsal lobes and apical hook directed dorsad.

**Female.** Unknown.

#### Etymology.

An adjective in nominative singular from “Hu-bei,” a province in China, referring to the type locality of this species.

#### Distribution.

This species has been found only at the type locality, Jing-shan County, Hu-bei Province, southcentral China, Oriental Region.

### Metalype shexianensis

Taxon classificationAnimaliaTrichopteraPsychomyiidae

Qiu & Morse sp. n.

http://zoobank.org/D13F26EB-54B7-41B2-AB94-AD067997D3ED

Fig. 3A–F

Metalype shexianensis Li, 1998: 221–222, figs 11.13–11.16, *nomen nudum*.

#### Type locality.

PRC, An-hui Province: She County, Yang-jia-tan, Feng-yuan-shui stream, 30°1.19'N; 118°17.84'E, 215 m, 24 May 1992, Collector JC Morse and Sun C-h; She County, Yan-yuan town, Huang-bai-shan village, Feng-yuan-shui stream, 30°5.94'N; 118°21.54'E, 717 m, 14 June 1991, collector Li Y-w.

#### Type specimen.

**Holotype.** Male, in 75% ethanol; head and prothorax, wings, cleared genitalia in different cotton-stoppered microvials inside one screwcap vial. Original label: “Ānhūi Shè-xiàn, Yáng-jiā-tán, Fēng-yuán-shǔi, 215 M elev., 24 May, 1992, Coll. Morse, Sun” “Metalype shexianensis, Holotype, Morse & Sun 1992”. Deposited in NJAU.

**Paratypes.** 2 males, in 80% ethanol, in cotton-stoppered microvial inside screwcap vial; one specimen cleared. Original label: “晚歙县岩源, 黄柏山村, 1991. 6-14” [Handwritten, Chinese characters = “Night She County Yan-yuan, Huang-bai-shan Village”] “Metalype shexianensis sp. n., Paratypes, Li & Morse 1996”. Deposited in CUAC.

#### Diagnosis.

This species resembles *Metalype anaktujuh* (Malicky, 1995b) (Malicky 1995b, page 23, figures in the top right corner) but can be distinguished by the following characters: (1) In lateral view the harpagones of *Metalype shexianensis* are slightly narrower in the middle than at the ends (Fig. 3A) in contrast to the harpagones of *Metalype anaktujuh* (Malicky 1995b, page 23, figure on the left); (2) In dorsal view the harpagones of *Metalype shexianensis* each bears two mesal processes, the anterior one is larger and truncate, the posterior one smaller and digitate (Fig. 3C),whereas the harpagones of *Metalype anaktujuh* each bears one truncate mesal process (Malicky 1995b, page 23, figure on the right).

#### Description.

**Male.** Forewings each 3.8–3.9 mm (n = 2). Compound eyes black, body yellow. Apicomesal spur of each hind tibia curved laterad and twisted apically, with two small subapical processes.

**Genitalia.** In dorsal view tergites IX+X widely separated from each other, each half triangular, in lateral view nearly L-shaped. In lateral view superior appendages setose, each wide at base, narrower at mid length than at the ends and digitate at apical half; in dorsal view mid length expanded mesally and covered with short setae; subapicomesal teeth each about two times as long as wide. In ventral view sternite IX slightly expanded posteriorly. In lateral view coxopodites triangular, in ventral view ovate and fused with each other for over half of their length. In lateral view harpagones slightly shorter than superior appendages, weakly sclerotized dorsally and tapered to apex, setose ventrally; in ventral view slightly expanded basomesally, curved mesad and slightly sclerotized at apices, each with two mesal processes subapically, anterior one larger; in mesal view truncate with notch, posterior one small, digitate, bearing few setae at apex. Phallobase expanded, phallicata narrow at base and slightly expanded at mid length, curved caudad for about 90° subapically beyond pair of short subapicodorsal lobes and apical hook directed dorsad.

**Female.** Unknown.

#### Etymology.

An adjective in nominative singular from “She-xian,” a county in An-hui Province, China, referring to the type locality of this species.

#### Distribution.

This species has been found only at the type localities in She County, An-hui Province, east central China, Oriental Region.

### Metalype truncata

Taxon classificationAnimaliaTrichopteraPsychomyiidae

Qiu & Morse sp. n.

http://zoobank.org/E51038FF-C1F6-4F79-9E2B-3F999021F30B

Fig. 4A–E

Metalype truncata Li, 1998: 221, figs 11.9–11.12, *nomen nudum*.

#### Type locality.

PRC, Si-chuan Province: Jiu-zhai-gou National Park, Jiu-zhai-gou County, 33°16.02'N; 103°55.08'E, 2000 m, 25 June 1990, Collector Chen Xiao-en (Chen X-e); Du-jiang-yan City, Qing-cheng mountain, Wei-jiang River, 32 km SW of Du-jiang-yan downtown, 30°53.90'N; 103°34.37'E, 930 m, 20 June 1990, Collector JC Morse, Yang L-f, Li Y-w and Chen X-e,

#### Type specimen.

**Holotype.** Male, in 75% ethanol, in cotton-stoppered microvial inside screwcap vial. Original label: “Sìchuān, Jiǔ-zhài-gōu, Nán-píng-xiàn, 2000 M elev., 25 June, 1990, Coll. Chen” “Metalype truncata, Holotype, Li & Morse 1996”. Deposited in NJAU.

**Paratypes.** 19 males, in 100% ethanol, one specimen in cotton-stoppered microvial with genitalia removed and cleared. Original label: “Sìchuān, Qīng-chéng-shān, 32 KM S.W. of Guàn xiàn, Wèi-jiāng-hé, 900 M elev., 27 June, 1990, Coll. Morse, Yang, Li, Chen” “Metalype truncata sp. n., Paratype, Li & Morse 1996” “Si-chuan Province P.R.C. Wei-jiang River Qin-cheng-shan, 32 km SW. of Du-jiang-yan City” [Handwritten]. Deposited in CUAC.

4 males, “Si-chuan Province P.R.C. Wei-jiang River Qin-cheng-shan, 32 km SW. of Du-jiang-yan City. Coll. Chen” [Handwritten]. Deposited in NJAU.

#### Diagnosis.

This species resembles *Metalype hubeiensis* sp. n. The differences are as detailed above for the latter species.

#### Description.

**Male.** Forewings each 3.9–4.5 mm (n = 10). Compound eyes black, body light brown. Apicomesal spur of each hind tibia truncate apically, with lobes on edge and central acute process.

**Genitalia.** In lateral view tergites IX+X slightly concave dorsally and acute at apex, in dorsal view each half round at apex. In dorsal view superior appendages setose, each with mesal setae short and apical setae thicker; in lateral view digitate, slightly curved caudad at mid length and gradually narrowed to blunt apex, in dorsal view subtriangular, each with subapicomesal tooth about 1.5 times as long as wide. In ventral view sternite IX slightly expanded posteriorly. In ventral view coxopodites ovate, fused for about half of their length, in lateral view triangular. In lateral view harpagones narrow at bases, gradually expanded to mid length, then narrowed abruptly, with dorsal surface of expanding area weakly sclerotized and slightly concave posteriorly; in ventral view harpagones hooked mesodorsad at apex, apex sclerotized and recurved anterad. Phallobase expanded, phallicata with small basoventral corner, then strongly sinuous and curved at mid length about 100°, with pair of wide subapicodorsal lobes, hooked about 170° dorsad apically.

**Female.** Unknown.

#### Etymology.

A Latin adjective in nominative singular, *truncata*, English “truncate,” referring to the apicomesal spur on each hind tibia.

#### Distribution.

This species has been found only in the type localities in Si-chuan Province, central China, Oriental Region.

### Metalype uncatissima

Taxon classificationAnimaliaTrichopteraPsychomyiidae

(Botosaneanu, 1970) new record

Fig. 5A–E

Psychomyia uncatissima Botosaneanu, 1970: 301–302. Type Locality: North Korea (Hamgjŏng-pukto); Levanidova et al. 1995: 7; Mey and Nozaki 2006: 24.
Metalype uncatissima (Botosaneanu, 1970): Li and Morse 1997: 274–275; Nozaki and Nakamura 2007: 94; Ivanov 2011: 191; Torii 2011: 7–12; Torii and Nakamura 2016: 425, 427, 429.

#### Material examined.

54 males, in 80% ethanol. One in cotton-stoppered microvial inside screwcap vial, with genitalia removed and cleared. Original label: “Heilongjiang, Shangzhixian, Maoershan-Town, Ashi River, Elev. 300 M, July 13, 1993, coll. Li Youwen & Sun Changhai” “Metalype uncatissima, (Botosaneanu)”. (50 in CUAC, 4 in NJAU).

#### Distribution.

This species has been reported from North Korea, Japan, and the Russian Far East. We report it now also from northeastern China (Hei-long-jiang Province), East Palearctic Region. The collection sites are: PRC, Hei-long-Jiang Province: Shang-zhi County, Mao-er-shan Town, A-shi River, 45°16.40'N; 127°30.26'E, 300 m, 13 July 1993, Collector Li Y-w and Sun C-h, 54 males (50 in CUAC, 4 in NJAU); Shang-zhi County, Wei-he Town, Yu-ling Tree Farm, close to Niu-shan Bridge, 44°39.33'N; 128°13.90'E, 380 m, 13 July 1993, coll. Li Y-w and Sun C-h, 1 male (NJAU); Tie-li City, Lang-xiang Town, Bei-lan River, Ba-lan Farm, 46°37.58'N; 129°7.29'E, 160m, 5 August 1993, coll. Li Y-w and Sun C-h, 2 males (NJAU); Wu-yi-lin Town, Yong-sheng, Wu-yun River, 48°37.09'N; 129°32.96'E, 160 m, 31 Jul. 1993, coll. Sun C-h, 4 males (NJAU).

**Figure 5.**
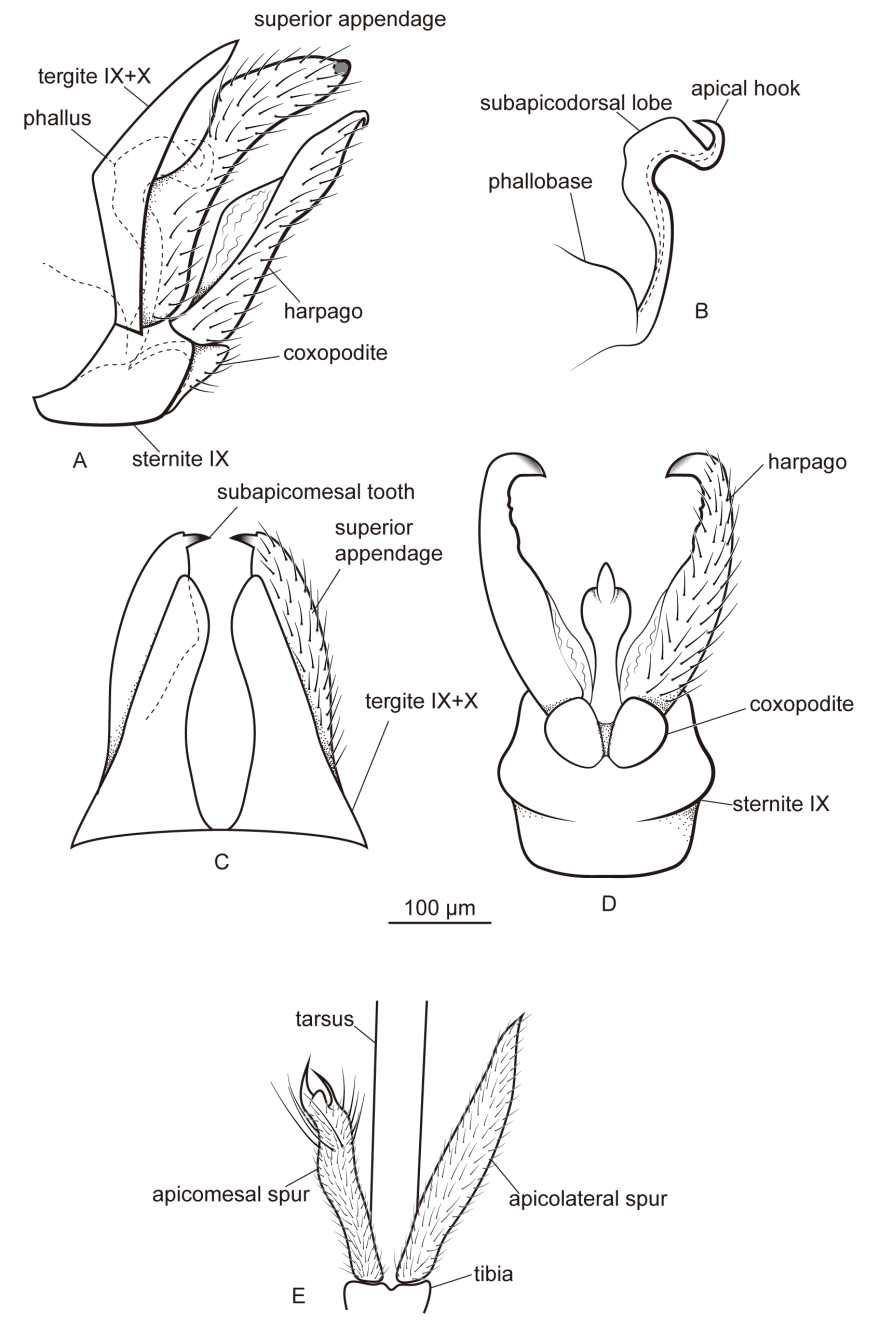
Male of *Metalype uncatissima* (Botosaneanu, 1970). **A** genitalia, left lateral **B** phallus, left lateral **C** genitalia, dorsal **D** genitalia, ventral **E** apical spurs of right hind leg, ventral.

In addition to the characters mentioned in the original description for this species (Botosaneanu 1970), the male apicomesal spur of each hind tibia is slightly twisted, bearing a transverse row of setae subapically (Fig. 5E); the apex has two acute processes and a short hump. The female was illustrated by Li and Morse (1997).

## Discussion

To date, only the characters of the type species, *Metalype fragilis*, have been used to diagnose the genus *Metalype*. Among the diagnostic characters now known to distinguish *Metalype* and *Paduniella*, synapomorphic characters for *Metalype* include the apicomesal spurs of the hind tibiae that are short and curved, twisted, truncate or forked apically; in the male genitalia the subapicomesal teeth of the superior appendages and the contorted phallus without a paramere. Synapomorphic characters for *Paduniella* include the 6-segmented maxillary palps, 4-segmented labial palps, and compressed male harpagones (Li and Morse 1997).

According to Li and Morse (1997, 1998), the most obvious differences between males of *Metalype* and *Psychomyia* are (1) The presence or absence of subapicomesal teeth on the superior appendages; (2) the size of the mesodorsal expansion of the basal half of each harpago; (3) the presence or absence of membranous basodorsal surfaces of the harpagones; and (4) the degree of fusion of male tergites XI+X with the superior appendages. These and other characters and their polarities are indicated in Table 2.

**Table 2.** Characters of selected *Psychomyia* species and all *Metalype* species, including species transferred here to *Metalype* (*). **Bold** character states are apomorphic. W = width, L = length.

Species	Male superior appendages subapicomesal teeth	Male harpagones expanded dorsally	Male harpagones membranous dorsally	Male hind tibiae apicomesal spurs length	Male hind tibiae apicomesal spurs shape	Male tergites IX+X fused with superior appendages	Female transverse row of setae on segment IX	Larval submental sclerites	Larval ventral apotome
*Metalype fragilis*	**with**	**yes**	**yes**	mesal>lateral	**curved**	no	**with**	W>L	W>5L
*Metalype anaktujuh*	**with**	**yes**	?	?	?	no	?	?	?
*Metalype hubeiensis*	**with**	**yes**	**yes**	mesal>lateral	**forked, curved**	no	?	?	?
*Metalype mahayinna*	**with**	**yes**	?	mesal>lateral	**truncate**	no	?	?	?
*Metalype shexianensis*	**with**	**yes**	**yes**	mesal>lateral	**forked, curved**	no	?	?	?
*Metalype truncata*	**with**	**yes**	**yes**	mesal>lateral	**truncate**	no	?	?	?
*Metalype uncatissima*	**with**	**yes**	**yes**	mesal>lateral	**forked, twisted**	no	**with**	W>L	W>5L
Metalype holzenthali*	**with**	**yes**	?	?	?	no	?	?	?
Metalype klapaleki*	**with**	**yes**	**yes**	mesal>lateral	**curved**	no	?	W>L	W>5L
Metalype kumari*	**with**	**yes**	?	?	?	no	?	?	?
Metalype nithaiah*	**with**	**yes**	**yes**	mesal>lateral	**truncate**	no	?	?	?
*Psychomyia flavida*	without	no	no	**lateral>mesal**	straight, acute	**yes**	without	**L>W**	**W<2L**
*Psychomyia pusilla*	without	no	no	**lateral>mesal**	straight, acute	**yes**	without	**L>W**	**W<2L**
*Psychomyia nomada*	without	no	no	**lateral>mesal**	straight acute	**yes**	?	**L>W**	**W<2L**

The presence or absence of subapicomesal teeth on the superior appendages is easily recognized. However, similar teeth are found in *Psychomyia amor* Malicky & Chantaramongkol, 1997; *Psychomyia amphiaraos* Malicky & Chantaramongkol, 1997; *Psychomyia andromache* Malicky, 1997; *Psychomyia andromeda* Malicky, 1997; *Psychomyia asvagosha* Schmid, 1961; *Psychomyia capillata* Ulmer, 1910; *Psychomyia dasaratha* Malicky, 1993b; *Psychomyia holzenthali* Schmid, 1997; *Psychomyia kalais* Malicky, 2004b; *Psychomyia kiskinda* Malicky & Chantaramongkol, 1993; *Psychomyia klapaleki*, *Psychomyia kumari* Schmid, 1997; *Psychomyia kuni* Malicky & Chantaramongkol, 1993; *Psychomyia lak* Malicky & Chantaramongkol, 1993; *Psychomyia monto* Malicky & Chantaramongkol, 1993; *Psychomyia neboissi* Schmid, 1997; *Psychomyia nithaiah*, *Psychomyia sinon* Malicky & Prommi, 2006; *Psychomyia sonlana* Oláh & Malicky, 2010; *Psychomyia vietnama* Oláh & Malicky, 2010; and *Psychomyia wigginsi* Schmid, 1997; Among them, *Psychomyia nithaiah*, *Psychomyia holzenthali*, *Psychomyia kumari*, and *Psychomyia klapaleki* are very similar to the three species transferred to *Metalype* by Li and Morse (1997): *Metalype anaktujuh*, *Metalype mahayinna* (Schmid, 1961), and *Metalype uncatissima*. The latter three species were included in Schmid’s *Psychomyia mahayinna* group together with *Metalype fragilis* (Schmid, 1997), so we hypothesize that these four species also belong to the genus *Metalype* and we cite them in *Metalype* through the remainder of this paper. All of the other 17 species above with subapicomesal teeth on the superior appendages are very different from *Metalype* by the following characters: (1) Tergites IX+X are fused with the superior appendages completely (synapomorphy; tergites IX+X are separated from the superior appendages in *Metalype*); (2) the superior appendages are greatly expanded basodorsally (synapomorphy; the superior appendages are not expanded in *Metalype*); (3) the superior appendages each have a large mesal concavity (synapomorphy; the superior appendages are without concavities in *Metalype*); (4) the coxopodites are semicircular (semicircular condition is synapomorphic; the coxopodites are round, triangular, or rectangular in *Metalype*); (5) the harpagones are forked (synapomorphy; the harpagones are single in *Metalype*).

The phallicata is more or less vertical basally and has a reversed-S-shape with an apical hook directed dorsad in *Metalype* species and all of these 17 *Psychomyia* species. This general shape is a synapomorphy for *Psychomyia* and *Metalype*, with the phallicata of *Psychomyia* species other than those 17 species generally more nearly horizontal and evenly curved, probably apomorphic within *Psychomyia*.

Moreover, *Psychomyia sonlana*, *Psychomyia sinon* and *Psychomyia andromache* also have a few more mesal spines on the superior appendages. Considering that there are many *Psychomyia* species with dense spines on the mesal surfaces of the superior appendages, it is possible that the subapicomesal teeth in these species are remnants or a modification of the mesal spines in one or more monophyletic groups within genus *Psychomyia* and thus these spines are a homoplasy, not homologous with the synapomorphic subapicomesal teeth of *Metalype*.

The peculiar shape of the expansion of the harpagones is not observed in *Psychomyia* species. It is not apparent also in *Metalype shexianensis* and *Metalype anaktujuh*. Instead, these two species have a mesal process on each of harpago, possibly representing a dorsal hump that shifted apicomesad. *Metalype holzenthali*, *Metalype klapaleki*, and *Metalype nithaiah* have that kind of expansion; whereas *Metalype kumari* has mesal processes that resemble those of *Metalype shexianensis* and *Metalype anaktujuh*. This expansion, possibly modified into a mesal process in some species, is likely a synapomorphy for some, if not all species of *Metalype*.

The membranous basodorsal surfaces of the harpagones are present in all *Metalype* specimens we observed, but this character is seldom mentioned in descriptions. Botosaneanu (1970) described this character in the original description of *Metalype uncatissima*. Malicky (1995a) mentioned this character in his re-description of *Metalype fragilis* and his description of *Metalype klapaleki*. Under the dissecting microscope, the membranous part is often without setae, the color is white or light yellow, the boundary between the membranous and the non-membranous parts is very obvious; after clearing, the membranous part is transparent and almost invisible, so that it can be distinguished from other parts. This character is likely a synapomorphy for *Metalype*.

Schmid (1997) described the separation of tergites IX+X and superior appendages as a character of his *Psychomyia mahayinna* group. This separation can be recognized in all *Metalype* species and the fusion of these structures is seen in most *Psychomyia* species. However, the fusion of tergites IX+X with the superior appendages is not very obvious in some *Psychomyia* species, for example *Psychomyia arefinae* Schmid, 1997; *Psychomyia schefterae* Schmid, 1997 and *Psychomyia scottae* Schmid, 1997. On the other hand, the bases of the superior appendages can be very wide (*Metalype anaktujuh*, *Metalype shexianensis*), which can make this character ambiguous. *Metalype holzenthali*, *Metalype klapaleki*, *Metalype kumari*, and *Metalype nithaiah* all have tergites IX+X separated from the superior appendages, as for other *Metalype* species. Thus the fusion of tergites IX+X and superior appendages seems to be a synapomorphy within genus *Psychomyia*.

**Figure 6.**
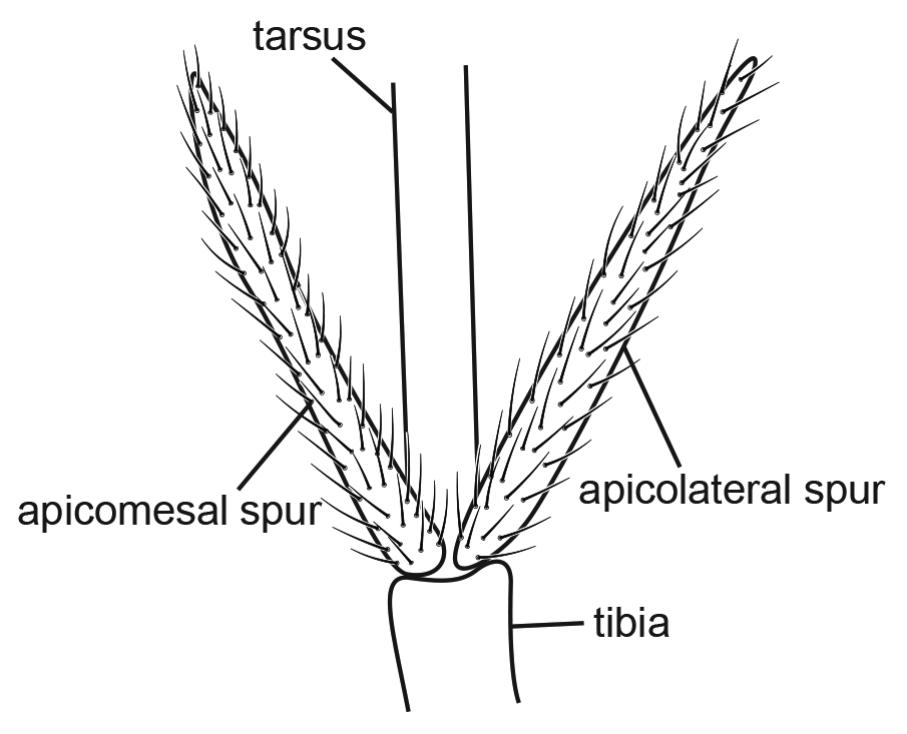
Male of *Metalype fragilis* (Pictet, 1834). Apical spurs of right hind leg, ventral.

**Figure 7.**
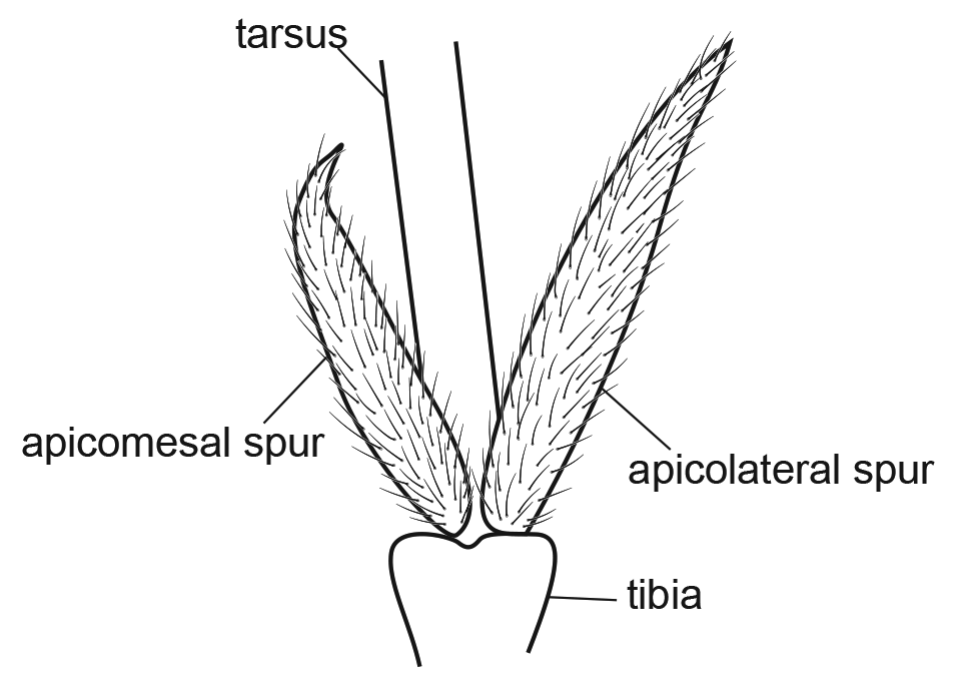
Male of *Metalype klapaleki* (Malicky, 1995a). Apical spurs of right hind leg, ventral.

The apicomesal spurs of hind tibiae on Psychomyiidae species other than those of *Metalype* are straight and acute. On all *Metalype* species we have studied, the apicomesal spurs are shorter than the apicolateral spurs, and these apicomesal spurs are more or less curved, twisted, truncate, or forked apically (Figs 2E, 3F, 4E, 5E, 6). All *Psychomyia* specimens we observed (including *Psychomyia flavida* Hagen, 1861; *Psychomyia extensa* Li, Sun, and Yang, 1999; *Psychomyia nomada* (Ross, 1938), and eleven unpublished species from China) have apicomesal spurs straight and slightly longer than the apicolateral spurs, never forked or truncate. *Metalype mahayinna* has apicomesal spurs similar to those of *Metalype truncata* (Malicky 1996; pers. comm). Males of *Metalype nithaiah* and *Metalype klapaleki* (Fig. 7) also have the apicomesal spur on each hind tibia shorter than the apicolateral spur and curved apically, supporting the hypothesis that these species belong in *Metalype*. The spurs of *Metalype holzenthali* and *Metalype kumari* are unknown to us. The slightly curved, twisted, forked or truncate apicomesal spurs on male hind tibiae is a synapomorphy within genus *Metalype*.

A difference between *Metalype* and *Psychomyia* females is that those of *Metalype* have a transverse row of setae on segment IX and those of *Psychomyia* species are without these setae (presence of the transverse setal row is synapomorphic). This difference is observed in females of *Psychomyia usuguronis* (Matsumura, 1931) (Ito et al. 2011), *Psychomyia flavida* (Ito et al. 2000), *Psychomyia pusilla* (Malicky 2004a), *Metalype fragilis* (Malicky 2004a), and *Metalype uncatissima* (Li and Morse 1997). However, the females are unknown for the four *Psychomyia* species we hypothesize here to belong to *Metalype* (*Metalype nithaiah*, *Metalype holzenthali*, *Metalype kumari*, and *Metalype klapaleki*).

Edington and Hildrew (1995) compared the larvae of *Psychomyia pusilla* and *Metalype fragilis*, and found three differences between them: (1) *Psychomyia pusilla* has the submental sclerites longer than wide, with dark patterns (synapomorphy); *Metalype fragilis* has the sclerites wider than long and without patterns. (2) *Psychomyia pusilla* has the ventral apotome small and triangular, no more than two times as wide as long (synapomorphy); *Metalype fragilis* has the ventral apotome expanded laterally, more than five times as wide as long. (3) *Psychomyia pusilla* has five or six teeth on the mesal surface of each anal claw; *Metalype fragilis* has two or three teeth (character polarity uncertain).

The long submental sclerite character is found in *Psychomyia flavida* and has been used for distinguishing the larvae of *Psychomyia* and *Paduniella*, with these sclerites wider than long in the latter (Wiggins 1996, Li and Morse 1997, 1998, Morse and Holzenthal 2008). We observed this long submental sclerite character for *Psychomyia nomada* specimens in the CUAC. On the other hand, the wide submental sclerites on larvae of *Metalype* species have been confirmed for the larvae of *Metalype fragilis* and *Metalype uncatissima* by many authors (Waringer and Graf 2011, Urbanič et al. 2003, Coppa et al. 2009, Torii 2011, Torii and Nakamura 2016). For all the species mentioned above, the small ventral apotome is usually coupled with the longer submental sclerites. One exception is the *Psychomyia* sp. larva from Aichi (Torii and Nakamura 2016); that larva has submental sclerites longer than their width, but the ventral apotome is wide. Dark patterns on the submental sclerites of *Psychomyia* are always present, although sometimes faint.

Coppa et al. (2009) concluded that the main character distinguishing the larva of *Paduniella vandeli* Decamps, 1965 from that of *Metalype fragilis* is the number of teeth on the ventral margin of each anal claw. The final instar larva of *Paduniella vandeli* bears seven or eight teeth on each anal claw (Coppa et al. 2009) while that of *Metalype fragilis* bears only two or three teeth (Coppa et al. 2009, Edington and Hildrew 1995). On the other hand, the larva of *Metalype uncatissima* has eight teeth on each anal claw (Torii 2011), *Paduniella nearctica* Flint, 1967 has four to six teeth; *Psychomyia flavida* (Morse and Holzenthal 2008) and *Psychomyia* sp. (probably *Psychomyia lumina*, Wiggins 1996) each have four teeth, and *Psychomyia nomada* has three or four teeth. The third instar larva of *Paduniella vandeli* also has three teeth on each anal claw (Coppa et al. 2009). Moreover, the teeth may not be uniform; some of them can be very small and hard to recognize. Thus, the number of teeth on each anal claw is not a reliable character for distinguishing the three genera.

Torii and Nakamura (2016) identified larvae of Psychomyiidae by molecular methods. They compared the morphological characters of larvae and noted that the episternum of each foreleg of *Metalype uncatissima* is without a vertical suture while larvae of *Paduniella horaiensis* Nishimoto, 2011 and *Psychomyia* sp. have the suture. We observed this suture on *Psychomyia nomada* specimens, but it is also present on the larva of *Metalype fragilis* (Coppa et al. 2009), so that the absence of the suture may be an autapomorphy of *Metalype uncatissima*. Torii and Nakamura (2016) also mentioned that the mature larva of *Metalype* (5–6 mm) is longer than the larva of *Paduniella* (3–4 mm). The phylogenetic evidence and diagnostic differences for larvae of *Metalype* and *Paduniella* remain inconclusive until more information on larvae is available.

The larva of *Metalype klapaleki* has submental sclerites wider than long. In fact, no differences have been found between larvae of *Metalype klapaleki* and larvae of *Metalype fragilis* (Urbanič et al. 2003), further supporting our hypothesis that *Metalype klapaleki* is a species of *Metalype*. Larvae are unknown for the other three species that we transfer here to *Metalype*. When they become known, we predict that the larval characters for those species will support our hypothesis.

## Conclusion

The male genitalia of *Metalype* and *Psychomyia* are very similar to each other, but there are some distinctive characters supporting the monophyly of each genus. The details are shown in Table 2. The known female genitalia and larvae of *Metalype* are similar to those of *Paduniella* and both of them are very different from female genitalia and larvae of *Psychomyia*. Treating *Metalype* as a synonym of *Psychomyia* may cause difficulties for identifying females and larvae of *Psychomyia*. However, female genitalia and larvae of only a few species are known in these genera, such that more information will be helpful. Based on the characters of males, we conclude that the following species should be transferred from *Psychomyia* to *Metalype*:

*Metalype holzenthali* (Schmid, 1997), comb. n.

*Metalype klapaleki* (Malicky, 1995a), comb. n.

*Metalype kumari* (Schmid, 1997), comb. n.

*Metalype nithaiah* (Malicky, 2014), comb. n.

### Key to males of *Metalype* species

**Table:** 

1	Superior appendages each with subapicomesal tooth and with tergites IX+X separated from superior appendages; hind tibiae each with apicomesal spur shorter than apicolateral spur and more or less curved, twisted, truncate, or forked apically	***Metalype***
–	Superior appendages usually without subapicomesal teeth and with tergites IX+X fused with superior appendages; hind tibiae each with apicomesal spur longer than apicolateral spur, straight, and acute apically	***Psychomyia***
2	Harpagones in ventral view each with large subapicomesal process, as large as apex (Fig. 3C)	**3**
–	Harpagones in ventral view without large subapicomesal processes (Figs 2D, 4D, 5D)	**5**
3	Harpagones in ventral view each with small mesal process behind larger mesal process (Fig. 3C)	***Metalype shexianensis***
–	Harpagones in ventral view without small mesal processes	**4**
4	Halves of tergites IX+X in dorsal view separated widely from each other, more than twice width of each base (Schmid 1997, fig. 17)	***Metalype kumari***
–	Halves of tergites IX+X in dorsal view separated narrowly from each other, separation about as much as width of each base (Malicky 1995b, page 23, figs on the top right corner)	***Metalype anaktujuh***
5	Harpagones in ventral view not hooked mesad apically (Schmid 1961, pl. 15, fig. 1)	***Metalype mahayinna***
–	Harpagones in ventral view each hooked mesad apically (Figs 2D, 4D, 5D)	**6**
6	Halves of tergites IX+X in dorsal view round apically (Fig. 4C)	**7**
–	Halves of tergites IX+X in dorsal view attenuate and blunt apically (Figs 2C, 5C)	**9**
7	Harpagones in ventral view each strongly hooked, with apices recurved anterad (Fig. 4D)	***Metalype truncata***
–	Harpagones in ventral view not as strongly hooked, with apices pointing mesad (Fig. 5D)	**8**
8	Harpagones in lateral view longer than superior appendages (Schmid 1997, fig. 12), Oriental Region	***Metalype holzenthali***
–	Harpagones in lateral view shorter than superior appendages (Malicky 1995a, fig. 1), West Palearctic Region	***Metalype klapaleki***
9	Halves of tergites IX+X in dorsal view curved slightly laterad apically (Malicky, 2014, pl. 8)	***Metalype nithaiah***
–	Halves of tergites IX+X in dorsal view not curved laterad apically (Figs 2C, 5C)	**10**
10	Superior appendages in dorsal view each strongly narrowed in apical half (Fig. 2C)	***Metalype hubeiensis***
–	Superior appendages in dorsal view not narrowed at apical half (Fig. 5C)	**11**
11	Harpagones in ventral view each expanded near bases (Fig. 5D), East Palearctic Region	***Metalype uncatissima***
–	Harpagones in ventral view expanded near mid length (Malicky 1995a, fig. 1), West Palearctic Region	***Metalype fragilis***

## Supplementary Material

XML Treatment for Metalype hubeiensis

XML Treatment for Metalype shexianensis

XML Treatment for Metalype truncata

XML Treatment for Metalype uncatissima
